# The high cost of care and limited evidence on cost-effective strategies for Lewy body dementia: systematic review of evidence

**DOI:** 10.1192/bjo.2023.626

**Published:** 2024-01-05

**Authors:** Erin Boland, Rachel Fitzpatrick, Dearbhail Ryan, Joseph Kane, Sara Betzhold, Iracema Leroi, Irina Kinchin

**Affiliations:** Trinity College Institute of Neuroscience, School of Medicine, Trinity College Dublin, Ireland; School of Medicine, Dentistry and Biomedical Sciences, Centre for Public Health, Queen's University Belfast, UK; Faculty of Health Sciences, Trinity College Dublin, Ireland; Trinity College Institute of Neuroscience, School of Medicine, Trinity College Dublin, Ireland; and Global Brain Health Institute, Trinity College Dublin, Ireland; Centre for Health Policy and Management, Trinity College Dublin, Ireland

**Keywords:** Clinical trial, core outcome set, cost of illness, economic evaluation, Lewy body dementia

## Abstract

**Background:**

Lewy body dementia (LBD) is a prevalent yet frequently underdiagnosed form of dementia, accounting for up to 15% of all dementia cases.

**Aims:**

This study aims to increase awareness and advocacy for LBD by gathering and critically assessing the economic evidence, including the cost of illness and cost-effectiveness of interventions for managing LBD.

**Method:**

A systematic literature review was undertaken with EMBASE, Medline, CINAHL, PsycINFO, NHS Economic Evaluation Database and EconLit. This search was supplemented by grey literature on Google Scholar and reviewing the reference lists of identified studies. The papers included in the review were published between 2008 and 2023, and involved participants with LBD (dementia with Lewy bodies or Parkinson's disease dementia), which either addressed the cost of illness or conducted an economic evaluation.

**Results:**

Thirteen papers were included, comprising ten cost-of-illness studies and three economic evaluations. The cost of LBD tends to be higher than that of other forms of dementia, such as Alzheimer's disease, and these costs escalate more steeply as the disease progresses. These cost differences may not be solely influenced by the subtype of dementia, but possibly also by patient characteristics like physical and cognitive abilities. Cost-effectiveness of potential interventions for LBD is limited.

**Conclusions:**

Despite numerous drug trials and other interventions for dementia, very few have targeted LBD, let alone explored the cost-effectiveness of such therapies for LBD. This disparity highlights the urgent need for cost-effective strategies and interventions targeting LBD. We propose the establishment of universally accepted standards for LBD research.

Lewy body dementia (LBD) is the second most common form of neurogenerative dementia after Alzheimer's disease. It refers to two related diagnoses, Parkinson's disease dementia (PDD) and dementia with Lewy bodies (DLB).^1–3^ LBD is estimated to account for up to 15% of all dementia cases in older adults.^4,5^ Given the prevalence and incidence of LBD, which is set to rise as the global populations age increases,^6^ diagnosis and management of the disease become increasingly important for healthcare services.

Managing the symptoms associated with LBD, including fluctuating cognitive impairment, parkinsonism, mood, hallucinations, autonomic dysfunction and sleep problems, can be clinically challenging and significantly affect the quality of life of those with LBD and their carers.^7^ Pharmacological management, however, is limited because patients have increased sensitivity to antipsychotics,^8–10^ and only one anti-dementia drug is licensed for PDD (not DLB). Antidepressants also are poorly tolerated,^11^ with no clear evidence of their effectiveness in treating mood in this population.^10,12^ Patients with motor issues resulting from Parkinson's disease have reduced treatment options because commonly prescribed anti-Parkinson's disease medications can exacerbate symptoms of psychosis and motor response is often poor.^7^ These factors affect treatment options and show a tailored and cost-effective treatment response is needed.

Studying the economic impact of LBD and identifying cost-effective strategies for its management is important for several reasons.^13^ First, such evidence provides a better understanding of the burden of disease on society, including financial costs to individuals, families and healthcare systems. Economic evidence can also raise awareness and advocacy for the disease, and promote policies that improve the lives of those affected by LBD. Additionally, a comparative economic analysis can help policy makers identify gaps in care and develop policies and programmes to address them. Finally, economic evidence can guide future research and investment in technologies for early diagnosis and management of LBD.

This literature review examines the existing economic evidence on the cost of illness and cost-effectiveness of interventions for managing LBD. By analysing the current literature, this review aims to provide evidence for whether there is, in fact, disparity in the health economic literature on dementia, help to inform service provision for LBD and provide critical directions to guide future LBD research.

## Method

### Protocol and registration

The systematic review was registered in the International Prospective Register of Systematic Reviews (PROSPERO) database (identifier CRD42022346808) and conformed to the evidence-based guidelines in the Preferred Reporting Items for Systematic Reviews and Meta-Analysis (PRISMA) guidelines.^14,15^

### Eligibility criteria and study selection

Using the PICO (Population, Intervention, Control and Outcomes) method, we searched for the cost of illness and economic evaluations of interventions targeting LBD. We excluded studies with no population of interest data, conference abstracts, posters and studies without full text available (Table 1).

**Table 1:** Inclusion and exclusion criteria

	Inclusion criteria	Exclusion criteria
Population	LBD (including DLB and PDD), all age groups and stages	No population of interest data
Design	Any design	
Intervention	Any intervention for LBD, DLB or PDD	
Outcome	Economic evaluation outcomes, cost-of-illness outcomes	Not an economic evaluation or cost of illness
Country/language	All countries/written in English	
Method	Primary studies	
Publication type	Published in peer-reviewed or grey literature	Conference abstracts, posters and studies without full text available
Year of publication	2008–2023	Before 2008

LBD, Lewy body dementia; DLB, dementia with Lewy bodies; PDD, Parkinson's disease dementia.

### Information sources

A search strategy was developed in consultation with an accredited librarian at Trinity College Dublin. The search was applied to six databases, including EMBASE, Medline, CINAHL, PsycINFO, NHS Economic Evaluation Database and EconLit. Additional grey literature was identified with Google advanced search. Beyond the initial search, we also conducted pearling by reviewing the reference lists of identified studies to ensure an exhaustive review of the literature in the field of study. The search was run on 16 July 2021, and updated on 6 March 2023. Data was extracted on 20 March 2023. A full search strategy is reported in Supplementary Appendix 1 available at https://doi.org/10.1192/bjo.2023.626.

### Selection and data collection process

Search results were imported into Covidence (Covidence, Melbourne, Australia; see https://www.covidence.org/). Two investigators independently applied the eligibility criteria and documented reasons for exclusion. Any discrepancies were flagged through the platform and resolved through discussion. The researchers independently extracted data from included studies into bespoke tables depending on the type of study, i.e. cost-of-illness or economic evaluation. Information was extracted from each included study on the country of origin, publication year, study size, costing as a primary focus (yes/no), epidemiological approach, method of resource quantification, study period and the cost reference year, perspective, study design, mean age of participants, setting, currency, cost category and cost components, main data source, the definition of LBD versus DLB and PDD, and disease assessment tools.

### Quality assessment in individual studies

Quality assessments were conducted independently by three investigators. The Larg and Moss checklist^16^ was used for the cost-of-illness studies, and the Drummond checklist^13^ for economic evaluation. The Larg and Moss checklist considers three key questions of study quality: What costs should have been measured? How well were resource use and productivity losses measured? And how well were the analysis and reporting performed? The Drummond checklist also assesses the quality of studies across three domains of study design, data collection methods, and analysis and interpretation of results. A quality score for each study was not created as these are not recommended because of weak reliability.^17^ Instead, the quality checklists are completed and presented in Supplementary Appendix 2.

## Results

### Overview

**Fig. 1:**
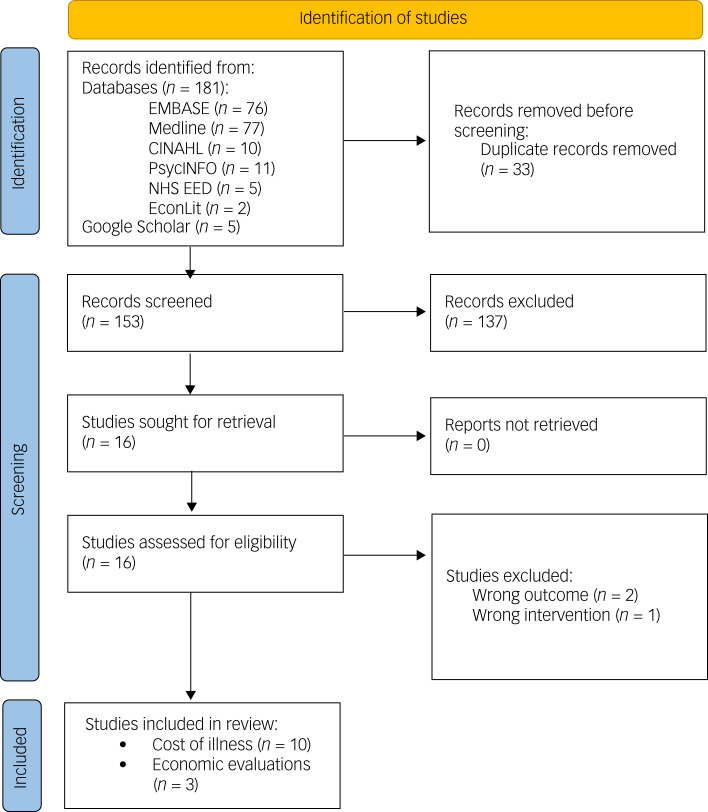
Preferred Reporting Items for Systematic Reviews and Meta-Analysis flow diagram. NHS EED, National Health Service Economic Evaluation Database.

The PRISMA flowchart in Fig. 1 outlines the process of identifying studies for review. The search identified 186 titles and abstracts that were screened based on the inclusion and exclusion criteria. In total, 13 full-text articles were analysed in this review, including ten cost-of-illness studies and three economic evaluations (refer to Supplementary Table 1 for a detailed description).

### Cost-of-illness studies

The cost-of-illness studies are reported in Table 2. Two of the ten studies focused exclusively on the costs associated with LBD.^18,19^ Six studies compared the costs of care between different subtypes of dementia,^20–22^ including three studies that examined costs related to LBD and Alzheimer's disease.^23–25^ Additionally, three studies assessed care costs for people with mild and/or moderate dementia.^26,27^

**Table 2:** Summary of the characteristics of included cost-of-illness studies (*n* = 10)

Characteristic	Number (%)	Reference
Country		
USA	4 (40%)	^19,21,23,24^
UK	2 (20%)	^20,27^
China	1 (10%)	^18^
Sweden, Finland, Norway	1 (10%)	^25^
France	1 (10%)	^22^
Norway	1 (20%)	^26^
Costing as a primary focus (yes)	10 (100%)	^18–27^
Study design		
Prospective	4 (40%)	^18,22,26,27^
Retrospective	6 (60%)	^19–21,23–25^
Epidemiological approach		
Prevalence	6 (60%)	^18,20,21,23–25^
Incidence	2 (20%)	^22,26^
Both	2 (20%)	^19,27^
Method of resource quantification		
Bottom-up	7 (70%)	^18,20,22–26^
Top-down	3 (30%)	^19,21,27^
Diagnostic criteria		
McKeith criteria	4 (40%)	^18,23–25^
Not specified	6 (60%)	^19–22,26,27^
Type of costs reported		
Direct	10 (100%)	^18–27^
Indirect	2 (20%)	^18,27^

#### Cost of LBD

Guo et al^18^ conducted a cost-of-illness study on the overall direct and indirect costs associated with LBD. Their findings indicated an annual cost of $21 378 for probable LBD in China (2021 US$). The team further broke down this cost into three categories: direct medical expenses, direct non-medical expenses and indirect expenses, which amounted to $3471, $3946 and $13 961, respectively. The study concluded that these total costs were significantly influenced by the activities of daily living (ADL) and the emotional stress experienced by the caregivers. They did not assess the costs associated with different forms of LBD, namely DLB and PDD.

Desai et al^19^ examined the financial burden associated with LBD in the USA (2019 US$). On average, patients with LBD incurred medical costs of $18 309 in the year before diagnosis. After diagnosis, the average medical costs for these patients rose to $29 174 in the first year and $22 814 in the fifth year. Hospital stays and out-patient visits were identified as the major contributors to these costs. Furthermore, they examined the financial burden of LBD when split into its two forms, and observed similar financial trends for DLB and PDD.

#### Cost comparison across dementia subtypes

In the USA, Murman et al^23^ compared the annual direct costs for patients with Alzheimer's disease and no Parkinsonism symptoms, patients with Alzheimer's disease and Parkinsonism symptoms and patients with DLB. They found that patients with Alzheimer's disease who also present with Parkinsonism symptoms, or have DLB, tend to face considerably higher healthcare costs compared with those only diagnosed with Alzheimer's disease. In comparison with the standard costs for Alzheimer's disease, the additional expenses for professional healthcare services significantly increased by an average of $7119 for patients with Alzheimer's disease and Parkinsonism symptoms and $13 754 for patients with DLB. When factoring in all medical and non-medical costs (total direct costs), these increased by $7394 for patients with Alzheimer's disease and Parkinsonism symptoms and $19 564 for patients with DLB. The study also showed that for every point increase on a scale measuring Parkinsonism symptom, there was a projected yearly rise of $784 in professional healthcare costs and $827 in total costs.

Likewise, Bostrom et al^25^ found that in Sweden, people with DLB had to spend a lot more on care each year compared with people with Alzheimer's disease. On average, those with DLB had to spend twice as much (€37 500 every year) than those with Alzheimer's disease (€18 200 per year). For people with DLB, functional impairment (as measured by ADL) correlated positively with cost of care. No relationship was observed between cognition (as measured by the Mini-Mental State Examination) or neuropsychiatric symptoms (as measured by the Neuropsychiatric Inventory) and cost of care.

Similar results were found when comparing costs for LBD (DLB and PDD) with other types of dementia. Henderson et al^20^ found that, in the 3 months before the study, the mean 3-month cost of professional care (paid care) in the UK was highest for people with PDD (£2001), which was more than double the cost for those with Alzheimer's disease (£852). With the exception of mixed dementia (£1256), the annual cost associated with DLB (£1026) was also higher than that of all other dementias (vascular dementia £890, frontotemporal dementia £1025).

When both professional care and unpaid care (such as that provided by family members) were considered, the cost was again highest for those with PDD at £8572, compared with £4618 for DLB, £3498 for Alzheimer's disease, £3773 for vascular dementia, £4337 for mixed dementia, £4783 for frontotemporal dementia and £5684 for other types of dementia.

A study by Dauphinot et al^22^ found that the cost of care in France in the first year after a patient's first visit to a memory clinic can vary depending on the type of dementia they have. Patients with LBD and Parkinson's disease had the highest costs. Although the average cost for any patient was €9885, those with Alzheimer's disease faced a slightly higher average cost of €10 444. However, the costs for those with LBD and Parkinson's disease were much higher, averaging €20 433 and €21 155, respectively. Dauphinot et al^22^ did not assess cost of care by LBD subtype.

Chen et al^21^ estimated the effect of dementia diagnosis subtype on direct healthcare costs and utilisation, using 2015 California Medicare fee-for-service data. From this enormous data-set, it was found that LBD was the most expensive dementia subtype, with a yearly cost of $22 514 per patient. Vascular dementia was the next most costly, with an annual cost of $21 002 per patient. Even after considering factors like each patient's demographic characteristics, comorbidities and duration of Medicare cover, LBD still remained the most expensive type of dementia. In comparison with people with Alzheimer's disease, those with LBD had healthcare costs that were 31% higher each year, and those with vascular dementia had costs that were 10% higher. Chen et al^21^ did not assess cost differences between LBD subtypes.

In contrast to these results, Zhu et al^24^ found no significant differences in the total cost and direct medical costs between patients with Alzheimer's disease and those with DLB in the USA. However, before adjusting for factors like age, gender, and mental and physical abilities, they found that patients with DLB had higher indirect costs, such as lost income owing to illness, but lower direct non-medical costs, such as those for personal care assistance, compared to patients with Alzheimer's disease. But when these factors were considered, the differences in costs between patients with Alzheimer's disease and those with DLB were no longer significant. This suggested that any perceived cost differences were mainly because of variations in the patients’ cognitive and functional status. Furthermore, Zhu et al^24^ included a smaller sample size than Chen et al,^21^ and included disproportionately more people with Alzheimer's disease (*n* = 170) than people with DLB (*n* = 25). So, although it initially appeared that there might be cost differences between Alzheimer's disease and DLB groups, once factors like age, gender, and mental and physical health were taken into account, these differences largely disappeared. However, Zhu et al^24^ also noted that their study included a small number of patients with DLB, which could have limited their ability to find real differences in the costs of care between the two groups.

#### Cost comparison by severity

Costs also seem to increase more steeply for LBD and its subtypes across the course of the disease. Henderson et al^27^ investigated the costs for people with mild-to-moderate dementia over three stages, or ‘waves’. They found that, total healthcare and social care costs for people with DLB were 1.5 times higher than for those with Alzheimer's disease, whereas the costs for people with PDD were three times as high. The study also found that the weekly cost of care increased more rapidly for people with DLB compared with those with Alzheimer's disease as the disease progressed.

Similarly, in Norway, Vossius et al^26^ conducted a prospective 3-year follow-up study and reported that it cost significantly more to care for someone with DLB (€3247 per month) than for someone with Alzheimer's disease (€1855 per month). The factors that seemed to have the biggest cost of care included place of living (e.g. own home or in a nursing home), whether they had a type of dementia other than Alzheimer's disease comorbidities and functioning. Individuals prescribed cholinesterase inhibitors (ChEIs), prescribed for dementia, experienced lower costs.

### Economic evaluations

Three economic evaluation studies were identified and summarised in Table 3. One was a full economic evaluation,^28^ and two were partial evaluations.^29,30^

**Table 3:** Summary of economic evaluation studies (*n* = 3)

Characteristic	Number (%)	Reference
Country		
UK	2 (66%)	^28,29^
Denmark	1 (33%)	^30^
Type of evaluation		
Full economic evaluation	1 (33%)	^28^
Partial economic evaluation	2 (66%)	^29,30^
Intervention		
Diagnostic and management toolkit	1 (33%)	^29^
Cholinesterase inhibitor intervention	1 (33%)	^28^
Psychosocial support	1 (33%)	^30^
Perspective		
UK National Health Service memory clinics	2 (66%)	^28,29^
Healthcare system	1 (33%)	^30^
Types of costs reported		
Direct and indirect	3 (100%)	^28–30^
Types of economic analysis		
Cost–utility analysis	1 (33%)	^28^
Cost–outcome description	1 (33%)	^29^
Cost analysis	1 (33%)	^30^
Types of outcomes		
Neuropsychiatric symptoms, activities of daily living, motor symptom, carer burden	1 (33%)	^29^
Informal care	1 (33%)	^30^
Quality of life, cognition	2 (66%)	^28,29^
Diagnostic accuracy	1 (33%)	^29^

### Full economic evaluations

#### ChEI intervention

Gustavsson et al^28^ conducted a cost–utility analysis comparing ChEI treatment in patients with mild-to-moderate Alzheimer's disease and DLB versus hypothetical matched controls. Patients with DLB responded better to ChEI treatment than patients with Alzheimer's disease, with lower cost per quality-adjusted life-year gained for treatment and a slight reduction in the cost of care in all three models used. The authors recommended the use of ChEI treatment in all patients with DLB.

### Partial economic evaluations

#### Management toolkit (DIAMOND-Lewy)

The DIAMOND-Lewy research programme^29^ conducted a partial economic evaluation of an intervention for patients with LBD (DLB and PDD). The intervention consisted of assessment and management toolkits for clinicians. Data were collected at baseline and at the 3- and 6-month follow-ups. Results from the pilot study showed improvements in global outcome measures, carer burden and depressive symptoms. Direct and indirect costs were measured, with health and social service use being the largest cost component. Total costs increased in the control arms between baseline and the 6-month follow-up and decreased in the intervention arms, with patients with DLB in the intervention arm showing a decrease in total cost. Quality-of-life data (as assessed by the EQ-5D-5L) showed small to modest, but consistent, decreases in mean and median scores for those with DLB. The authors concluded that with such small samples, data were insufficient to draw robust conclusions on the impact of the intervention on costs and quality of life.

#### Psychosocial intervention

Søgaard et al^30^ conducted a 3-year cost analysis of a psychosocial intervention delivering counselling and educational sessions for patients with Alzheimer's disease and LBD and their caregivers. The study included 330 patient–caregiver dyads, of which nine included a person with LBD. The costs assessed were healthcare resource use, informal care and productivity loss of the caregiver. The intervention group had lower costs in formal healthcare and nursing homes, but higher use of informal care compared with controls, resulting in an average cost of €3401 higher than control. The authors concluded that early psychosocial intervention in Alzheimer's disease could be cost-saving from a formal healthcare perspective, but may burden the caregiver more than it saves costs in an informal, broader societal perspective. No LBD-specific economic outcomes were reported.

### Quality assessment

#### Cost-of-illness studies

The quality of the ten cost-of-illness studies was assessed with the Larg and Moss checklist, included in Supplementary Table 2. The ten studies had varied levels of quality. Regarding the analytical framework employed, six studies^18,20–23,27^ met all six criteria. Regarding methodology and data, most studies did not meet the criteria, with the exception of one study^25^ that met all nine criteria. This suggests a general need for improvement in methodological rigor and data handling. Finally, in the area of analysis and reporting, none of the studies fulfilled all the requirements, highlighting a significant gap in comprehensive and transparent reporting practices.

#### Economic evaluation studies

The quality of the three economic evaluation studies was assessed with the Drummond checklist in Supplementary Table 3. The one full economic evaluation^28^ covered all of the quality criteria in study design. However, for data collection, it met only eight out of 14 quality criteria. In terms of the quality of analysis and reporting of results, the study met seven out of 13 of the criteria. This indicates that although Gustavsson et al^28^ is the most methodologically high-quality economic evaluation among the three studies in this review, there are still areas, particularly in data collection and analysis/reporting, where it could be enhanced. Partial evaluations were assessed for quality, but as they do not have all components of economic evaluations, are not compared here.

## Discussion

This systematic review evaluated the cost of LBD and the cost-effectiveness of potential interventions for managing LBD by assessing 13 studies, of which ten reported the cost of illness and three conducted economic evaluations of interventions targeting LBD. Evidence suggests that the cost of LBD is substantial and often higher than other forms of dementia. The costs vary by location and can be influenced by a range of factors, including the patient's level of independence in ADL and the emotional stress of caregivers. Studies in China and the USA indicate annual costs for LBD ranging from $21 378^18^ to $29 174,^19^ respectively. These include medical, non-medical and indirect costs such as caregiver stress and loss of income.

When comparing LBD with Alzheimer's disease, studies show costs can be significantly higher for LBD.^20,23,25^ The cost for LBD also appears to increase more significantly as the disease progresses. A study from the UK found that the costs for individuals with LBD escalated faster than those for individuals with Alzheimer's disease.^27^ This was also observed in a Norwegian-based cohort study.^26^

In addition, three other studies provided information about the cost-effectiveness of interventions for managing LBD. The DIAMOND-Lewy project demonstrated that providing clinicians with specialised toolkits for assessing and treating patients yielded positive results.^29^ These toolkits may lead to better patient outcomes, less strain on caregivers, fewer symptoms of depression and lower overall costs for patients with LBD.^29^ Anti-dementia drug treatment in the form of ChEIs also showed good value for money for people with DLB and reduced the cost of care.^28^ Finally, another study found that providing extra support and counselling to patients with Alzheimer's disease and DLB and their caregivers resulted in slightly higher average costs, but led to less use of professional healthcare services and nursing homes. Instead, patients were more likely to receive care from their friends and family.^30^ Overall, although these studies yield promising results, they are limited in number, and highlight the need for further interventions specifically designed to meet the unique needs of people with LBD.

This study offers valuable insights, but it is important to acknowledge some of its limitations. Because of the scarcity of economic evaluations specifically focused on LBD, definitive conclusions cannot be drawn. The differences in types of costs included, outcomes and interventions considered, and the general methodologies used to present economic data made direct comparisons between these studies challenging. This wide variation hindered our ability to synthesise the results, including the possibility of conducting a meta-analysis. Moreover, in referring to dementia types, we adopted the terminology used in the papers being reviewed. This approach had its limitations, as it was unclear in some studies whether they were addressing LBD as a whole or solely the DLB subtype, given the terms LBD and DLB are often used interchangeably. Since we had no means to verify which term was intended, this added to the difficulty in comparing studies.

### Implications

Despite numerous drug trials and other interventions for dementia, very few have targeted LBD, let alone explored the cost-effectiveness of such therapies for LBD. Moreover, the review revealed a pressing need for standardisation in clinical trials and health economics studies associated with LBD. This encompasses the need for a uniform approach to defining outcomes and nomenclature, which would enhance comparability across studies, thereby facilitating more conclusive interpretations of the research.

One potential strategy to address these issues involves developing a consensus driven by diverse stakeholders. This approach would convene field experts and those directly affected by LBD and other forms of dementia to agree upon a universal set of standards for conducting and reporting research in this area. Implementing such standards could significantly elevate the quality and comparability of research, fostering better understanding and management of these conditions. Our team is presently undertaking a Delphi study to create a core outcome set standard for LBD, marking a significant step toward these goals.^6^

Although the evidence points towards higher costs of LBD, the review also highlighted a disproportionate scarcity of economic evaluations of interventions aimed explicitly at managing LBD. Compared with other forms of dementia, such as Alzheimer's disease and vascular disease, LBD appeared to be underrepresented in economic research. Cost-effectiveness analyses are critical for clinical trials to ensure new therapies can be adequately evaluated by health technology agencies. Such research can foster informed clinical decision-making, prompt the development of cost-effective care strategies and, ultimately, enhance the quality of life for people living with LBD.

## Supporting information

Boland et al. supplementary material Boland et al. supplementary material

## Data Availability

The data that support the findings of this study are available from the corresponding author, I.K., on reasonable request.
