# Supplementary material for: The high cost of care and limited evidence on cost-effective strategies for Lewy body dementia: systematic review of evidence
Source: BJPsych Open. 2024 Jan 5;10(1):e20. doi: 10.1192/bjo.2023.626 (PMC10790215; doi:10.1192/bjo.2023.626)
Supplement: Boland et al. supplementary material [file S2056472423006269sup001.docx]

# Appendix A

**Search strategy**

### EMBASE [7 ']

'economic evaluat’on'/ex‘

'economic evaluati’n*':ti,‘b

'cost effectiveness analy’is'/exp '

('cost effectiven’ss' 'R 'cost effectiveness analy'?s' 'R 'cost effectiveness rat'o*' 'R 'cost efficiency analy'?s'):ti, 'b

'cost utility analy'is'/ex '

('cost util'ty' 'R 'cost utility analy'?s'):ti, 'b

'cost benefit analy’is'/exp '

('cost analy'?s' 'R 'cost bene’it' 'R 'cost benefit analy'?s' 'R 'cost benefit rat'o*' 'R 'cost-benefit analy'?s'):ti, 'b

'cost consequence analy’is'/ex '

('cost consequen'e*' 'R 'cost consequence analy'?s'):ti, 'b

'cost minimization analy’is'/exp

cost-minimi?ation:ti, 'b

'return on investm'nt'/e'p

'return on investme’t*':ti,ab

#1 OR #2 OR #3 OR #4 OR #5 OR #6 OR #7 OR #8 OR #9 OR #10 OR #11 OR #12 OR #13 OR # '4

'diffuse Lewy body dise'se'/ex '

('’lb' 'R 'd'bd' 'R '’bd' 'R 'lewy body dement'a*' 'R 'lewy body disea'e*' 'R 'dementia with lewy b'd*' 'R 'diffuse lewy body disea'e*'):ti,ab

#16 OR #17

#15 AND #18

### Medline [77]

economics/

value of life/

e "p "costs and cost analy" is"/

exp economics, hospital/

exp economics, medical/

economics, nursing/

economics, pharmaceutical/

e "p "fees and char" es"/

exp budgets/

budget*.ti,ab.

cost*.ti.

(economic* or pharmaco?economic*).ti.

(price* or pricing*).ti,ab.

(cost* adj2 (effective* nswerlit* or benefit* or minimi* or unit* nsweromat* or variable*)).abnsweranc* or fee or fees).ti,ab.

(value adj2 (money or monetary)).ti,ab.

or/1-16

Lewy Body Disease/

(dlb OR dlbd OR lbd OR lewy body dementia* OR lewy body disease* OR dementia with lewy bod* OR diffuse lewy body disease*).ti,ab.

or/18-19

17 AND 20

### CINAHL [10]

("H "Cost Benefit Analy" is")

TI ("economic evaluation*") OR AB ("economic evaluation*")

TI ("Cost consequence analys*" OR "cost-effectiveness") OR AB ("Cost consequence analys*" OR "cost-effectiveness")

TI ("cost effective" OR "cost benefit*") OR AB ("cost effective" OR "cost benefit*")

TI ("cost utility") OR AB ("cost utility")

TI ("cost minimi?ation analysis") OR AB ("cost minimi?ation analysis")

S1 OR S2 OR S3 OR S4 OR S5 OR S6

(“H "Lewy Body Dise”se")

TI (dlbd OR lbd OR "lewy body dementia*" OR "lewy body disease*" OR "dementia with lewy bod*" OR "diffuse lewy body disease*") OR AB (dlbd OR lbd OR "lewy body dementia*" OR "lewy body disease*" OR "dementia with lewy bod*" OR "diffuse lewy body disease*")

S8 OR S9

S7 AND S10

### PSYCINFO [11]

"E "Costs and Cost Analy" is"

TI ("economic evaluation*") OR AB ("economic evaluation*")

TI ("Cost consequence analys*" OR "cost-effectiveness") OR AB ("Cost consequence analys*" OR "cost-effectiveness")

TI ("cost effective" OR "cost benefit*") OR AB ("cost effective" OR "cost benefit*")

TI ("cost utility") OR AB ("cost utility")

TI ("cost minimi?ation analysis") OR AB ("cost minimi?ation analysis")

S1 OR S2 OR S3 OR S4 OR S5 OR S6

"E "Dementia with Lewy Bod" es"

TI (dlbd OR lbd OR "lewy body dementia*" OR "lewy body disease*" OR "dementia with lewy bod*" OR "diffuse lewy body disease*") OR AB (dlbd OR lbd OR "lewy body dementia*" OR "lewy body disease*" OR "dementia with lewy bod*" OR "diffuse lewy body disease*")

S8 OR S9

S7 AND S10

### NHS EED [5]

MeSH Search: Lewy Body Disease

### EconLit [2]

TI ("economic evaluation*") OR AB ("economic evaluation*")

TI ("Cost consequence analys*" OR "cost-effectiveness") OR AB ("Cost consequence analys*" OR "cost-effectiveness")

TI ("cost effective" OR "cost benefit*") OR AB ("cost effective" OR "cost benefit*")

TI ("cost utility") OR AB ("cost utility")

TI ("cost minimi*ation analysis") OR AB ("cost minimi*ation analysis")

S1 OR S2 OR S3 OR S4 OR S5

TI (dlbd OR lbd OR "lewy body dementia*" OR "lewy body disease*" OR "dementia with lewy bod*" OR "diffuse lewy body disease*") OR AB (dlbd OR lbd OR "lewy body dementia*" OR "lewy body disease*" OR "dementia with lewy bod*" OR "diffuse lewy body disease*")

S7 OR S8

**Appendix B**

**Table B. 1. Summary of economic evaluation studies**

| **Lead author (year)** | **Objective** | **Country (currency)** | **Study population** | **LBD diagnosis criteria** | **Study design** | **Year of pricing** | **Time horizon** | **Direct costs** | **Indirect Costs** | **Source of effectiveness data** | **Link between effectiveness and cost data** | **Sensitivity analysis** | **Conclusion** |
| --- | --- | --- | --- | --- | --- | --- | --- | --- | --- | --- | --- | --- | --- |
| Gustavsson et al. (2009)^46^ | To assess the cost effectiveness of ChEI treatment for AD and DLB | GBP | n= 1506 (LBD = 163; DLB = 112) assumed baseline age and sex derived from SATS study (74 years, male 35%). Mean age not provided (79.9 (SD 7.2) for all diagnoses from Van Der Putt study data. | Based on clinici’ns' judgement. | 4-month open label follow-up data | 2001 to 2005 | 5 years | direct and indirect medical costs, full time care, no informal care costs included | None included | Data from Van der Putt et al., (2006) and Wallin et al., (2007). Mean MMSE 20.3 at baseline. 40/112 considered "moderate" severity | 3 models: SHTAC model; micro-simulation model; Markov model | None | The cost of care was reduced by treatment in all three models. Moderate DLB was most cost-effective group; sufficient to recommend use in routine practice. |
| Søgaard et al. (2014)^48^ | To investigate the impact of a psychosocial intervention for patients with AD and LBD and their caregiver on resource use and costs | Euro | 330 dyads of patients and caregivers. LBD diagnosis (2.7%) intervention n = 7, control n = 2. Mean patient age 76. % female: intervention 53% patients and 67% caregivers; control 55% patients and 66% caregivers. | McKeith criteria | Randomized controlled trial | Not mentioned | 3 years | Resource use in primary and secondary health care sectors (arising from both dementia and its comorbidities) for both patients and caregivers | Informal care, production loss | Single study | Case reports, registers of National Board of Health, Resource Utilisation in Dementia questionnaire | Assessed impact of alternative tinsweronons. Informal care is sensitive to the choice of time/cost estimate for caregivers, which drives the overall estimate for the cost. | The intervention may burden the caregiver more than it saves costs in formal health care. No LBD specific conclusions reported. |
| O'Brien et al. (2021) ^47^ | To determine the feasibility of a cluster RCT of clinical toolkits for assessing and managing DLB and PDD | GBP | n=109; DLB = 77; mean age control 77, intervention 79; female: 19.2% control, 22.7% intervention | Based on clinici’ns' judgement | Pilot randomized controlled trial | 2017 to 2018 | 6 month follow up | health and social service cost which made up most of the total costs, medication costs which was found to increase slightly for both intervention and control, and intervention delivery costs. | Private healthcare costs and lost earning potential for time spent attending appointments, for both patient and caregiver. | EQ-5D-5L questionnaire, ONS data | data from same sample as used in effectiveness analysis. | None | Changes in QALYs in each arm were small, but with consistent decreases in mean and median scores for those with DLB, indicating a benefit for those in the intervention arm. |

**Table B. 2. Quality assessment of the cost-of-illness studies using the Larg and Moss checklist**

|  | **Bostrom** | **Chen** | **Henderson** | **Murman** | **Vossius** | **Zhu** | **Guo** | **Dauphinot** | **Desai** | **Henderson** |
| --- | --- | --- | --- | --- | --- | --- | --- | --- | --- | --- |
| **1. ANALYTICAL FRAMEWORK: WHAT COSTS SHOULD HAVE BEEN MEASURED?** | | | | | | | | | | |
| **(a) What was the motivation and perspective of the study?** |  |  |  |  |  |  |  |  |  |  |
| **(b) Was the appropriate epidemiologic approach taken?** |  |  |  |  |  |  |  |  |  |  |
| **(c) Was the study question well specified?** |  |  |  |  |  |  |  |  |  |  |
| (i) Were all relevant, non-trivial cost components and their stakeholders identified? |  |  |  |  |  |  |  |  |  |  |
| (ii) Were necessary timeframes specified? |  |  |  |  |  |  |  |  |  |  |
| (iii) Was a case of disease or risk factor adequately and appropriately defined? |  |  |  |  |  |  |  |  |  |  |
| (iv) Was the counterfactual population occurrence plausible and meaningful? |  |  |  |  |  |  |  |  |  |  |
| **2. METHODOLOGY AND DATA: HOW WELL WERE RESOURCE USE AND PRODUCTIVITY LOSSES MEASURED** | | | | | | | | | | |
| **(a) Was an appropriate method(s) of quantification used, such that** |  |  |  |  |  |  |  |  |  |  |
| (i) additional, or excess, costs were measured? |  |  |  |  |  |  |  |  |  |  |
| (ii) only costs specific to (caused by) the health problem were included (confounders controlled)? |  |  |  |  |  |  |  |  |  |  |
| (iii) all important effects were captured? |  |  |  |  |  |  |  |  |  |  |
| (iv) important differences across subpopulations were accounted for? |  |  |  |  |  |  |  |  |  |  |
| (v) the required level of detail could be provided? |  |  |  |  |  |  |  |  |  |  |
| **(b) Was the resource quantification method(s) well executed?** |  |  |  |  |  |  |  |  |  |  |
| (i) For population-based studies, were cost allocation methods, data and assumptions valid? |  |  |  |  |  |  |  |  |  |  |
| (ii) For person-based studies, were appropriate statistical tests performed and reported? |  |  |  |  |  |  |  |  |  |  |
| (iii) Were data representative of the study population? |  |  |  |  |  |  |  |  |  |  |
| (iv) Were there any other relevant resource quantification issues? |  |  |  |  |  |  |  |  |  |  |
| **(c) Were healthcare resources valued appropriately?** |  |  |  |  |  |  |  |  |  |  |
| **(d) Was the approach for valuing production losses justified, and assumptions valid?** |  |  |  |  |  |  |  |  |  |  |
| **(e) Was the inclusion of intangible costs appropriate:** |  |  |  |  |  |  |  |  |  |  |
| (i) Was double counting of mortality-related production losses avoided |  |  |  |  |  |  |  |  |  |  |
| (ii) Were losses valued appropriately, given the study's perspective? |  |  |  |  |  |  |  |  |  |  |
| **(3) ANALYSIS AND REPORTING** | | | | | | | | | | |
| **(a) Did the analysis address the study question?** |  |  |  |  |  |  |  |  |  |  |
| **(b) Was a range of estimates presented?** |  |  |  |  |  |  |  |  |  |  |
| **(c) Were the main uncertainties identified?** |  |  |  |  |  |  |  |  |  |  |
| **(d) Was a sensitivity analysis performed on:** |  | | | | | | | | | |
| (i) important (uncertain) parameter estimates? |  |  |  |  |  |  |  |  |  |  |
| (ii) key assumptions? (including the counterfactual) |  |  |  |  |  |  |  |  |  |  |
| (iii) point estimates? (based on confidence or credible intervals) |  |  |  |  |  |  |  |  |  |  |
| **(e) Was adequate documentation and justification given for cost components, data and sources, assumptions and methods?** |  |  |  |  |  |  |  |  |  |  |
| **(f) Was uncertainty around the estimates and its implications adequately discussed?** |  |  |  |  |  |  |  |  |  |  |
| **(g) Were important limitations discussed regarding the cost components, data, assumptions and methods?** |  |  |  |  |  |  |  |  |  |  |
| **(h) Were the results presented at the appropriate level of detail to answer the study question (cost components; disease subtypes, severity, stage; subpopulation groups, cost bearers)?** |  |  |  |  |  |  |  |  |  |  |

**Table B. 3. Quality assessment of included economic evaluations using the Drummond checklist**

|  | **Gustavsson et al. (2009)^46^** | **Søgaard et al. (2014)^48^** | **O’Brien et al. (2021) ^47^** |
| --- | --- | --- | --- |
| **Study design** |  |  |  |
| 1. Was the research question stated? |  |  |  |
| 2. Was the economic importance of the research question stated? |  |  |  |
| 3. Was/were the viewpoint(s) of the analysis clearly stated and justified? |  |  |  |
| 4. Was a rationale reported for the choice of the alternative programmes or interventions compared? |  |  |  |
| 5. Were the alternatives being compared clearly described? |  |  |  |
| 6. Was the form of economic evaluation stated? |  |  |  |
| 7. Was the choice of form of economic evaluation justified in relation to the questions addressed? |  |  |  |
| **Data collection** |  |  |  |
| 8. Was/were the source(s) of effectiveness estimates used stated? |  |  |  |
| 9. Were details of the design and results of the effectiveness study given (if based on a single study)? |  |  |  |
| 10. Were details of the methods of synthesis or meta-analysis of estimates given (if based on an overview of a number of effectiveness studies)? |  |  |  |
| 11. Were the primary outcome measure(s) for the economic evaluation clearly stated? |  |  |  |
| 12. Were the methods used to value health states and other benefits stated? |  |  |  |
| 13. Were the details of the subjects from whom valuations were obtained given? |  |  |  |
| 14. Were productivity changes (if included) reported separately? |  |  |  |
| 15. Was the relevance of productivity changes to the study question discussed? |  |  |  |
| 16. Were quantities of resources reported separately from their unit cost? |  |  |  |
| 17. Were the methods for the estimation of quantities and unit costs described? |  |  |  |
| 18. Were currency and price data recorded? |  |  |  |
| 19. Were details of price adjustments for inflation or currency conversion given? |  |  |  |
| 20. Were details of any model used given? |  |  |  |
| 21. Was there a justification for the choice of model used and the key parameters on which it was based? |  |  |  |
| **Analysis and interpretation of results** |  |  |  |
| 22. Was time horizon of cost and benefits stated? |  |  |  |
| 23. Was the discount rate stated? |  |  |  |
| 24. Was the choice of rate justified? |  |  |  |
| 25. Was an explanation given if cost or benefits were not discounted? |  |  |  |
| 26. Were the details of statistical test(s) and confidence intervals given for stochastic data? |  |  |  |
| 27. Was the approach to sensitivity analysis described? |  |  |  |
| 28. Was the choice of variables for sensitivity analysis justified? |  |  |  |
| 29. Were the ranges over which the parameters were varied stated? |  |  |  |
| 30. Were relevant alternatives compared? |  |  |  |
| 31. Was an incremental analysis reported? |  |  |  |
| 32. Were major outcomes presented in a disaggregated as well as aggregated form? |  |  |  |
| 33. Was the answer to the study question given? |  |  |  |
| 34. Did conclusions follow from the data reported? |  |  |  |

*green= yes, orange = n/a, red = no
